# Coronavirus Disease 2019 Associated Acute Myocardial Infarction and Atrial Fibrillation: A Case Report

**DOI:** 10.7759/cureus.31216

**Published:** 2022-11-07

**Authors:** Henry Mann, Aysham Chaudry, Tsering Dolkar, Alix Dufresne

**Affiliations:** 1 Internal Medicine, One Brooklyn Health System/Interfaith Medical Center, Brooklyn, USA; 2 Medical Student, Touro College of Osteopathic Medicine, Middletown, USA; 3 Cardiology, One Brooklyn Health System/Interfaith Medical Center, Brooklyn, USA

**Keywords:** acute hypoxic respiratory failure, nstemi, new onset afib, coronavirus 19, myocardial infarction, atrial fibrillation

## Abstract

In the last two years since the inception of the Coronavirus pandemic, there have been a myriad of reports and studies related to Coronavirus disease 2019 (COVID-19). We present a unique case of COVID-19 associated with both acute myocardial infarction and new-onset atrial fibrillation (AFIB) in an elderly lady which is the first reported case to the best of our knowledge. The patient was symptomatic with acute COVID-19 and developed a type 2 myocardial infarction with new-onset AFIB. The patient also developed sepsis which may have contributed to the development of AFIB.

## Introduction

COVID-19 is known to primarily affect the pulmonary system, however cardiovascular complications of COVID-19 including acute myocardial infarction and arrhythmias are well described [1]. According to one article, the incidence of new onset AFIB in patients with COVID-19 is 3.6-6.7% [2]; according to another study that used the results from the American Heart Association’s COVID-19 Cardiovascular registry, the incidence was 5.4% [3]. A meta-analysis found the prevalence of acute myocardial infarction in patients with COVID-19 infection to be 20% [4]. Here, we present a case of new-onset AFIB and acute myocardial infarction in a 72-year-old female patient who presented with altered mental status secondary to COVID-19 pneumonia; this is the only case report to our knowledge documenting this.

## Case presentation

**Figure 1:**
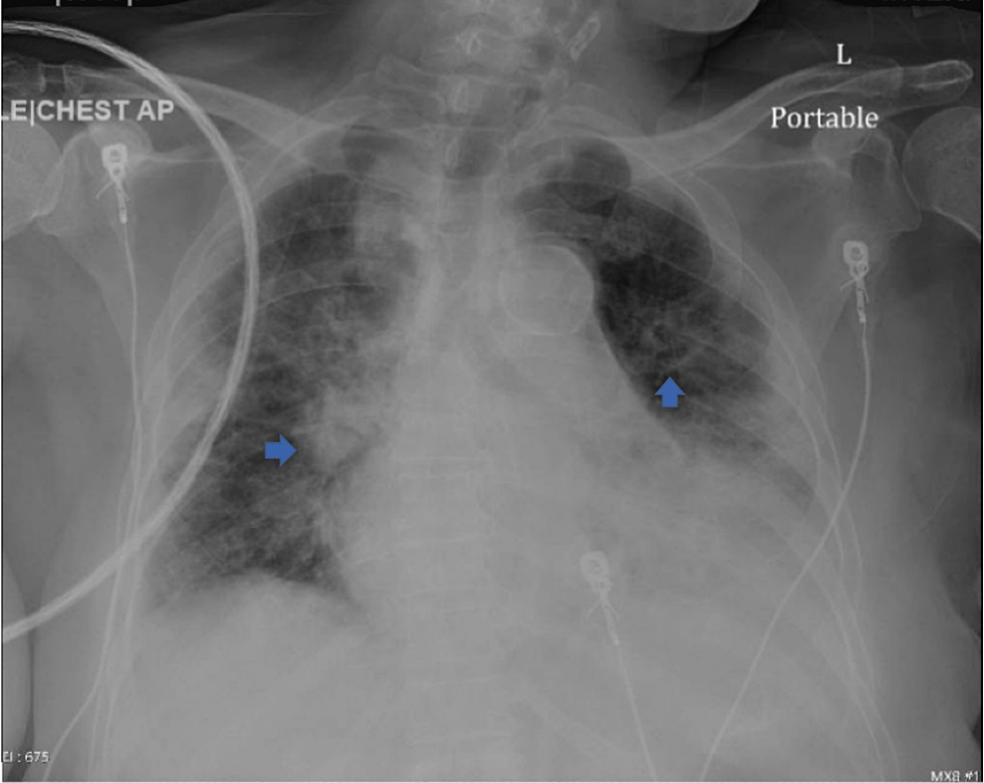
Chest X-ray on Admission Chest X-ray reveals diffuse bilateral airspace opacities consistent with bilateral pneumonia.

**Figure 2:**
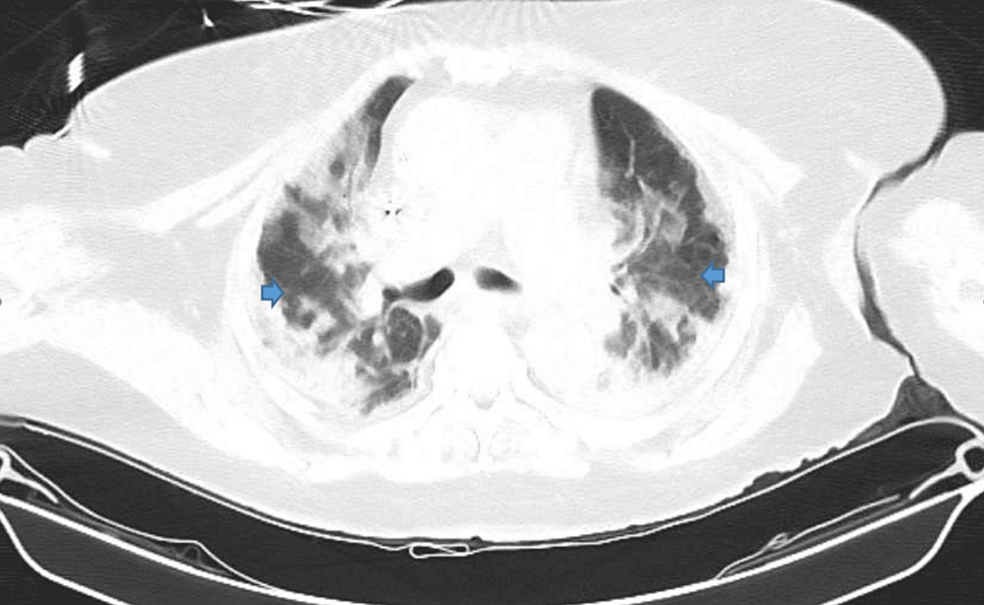
Chest CT on Admission Multifocal consolidation with ground glass opacities are seen.

**Figure 3:**
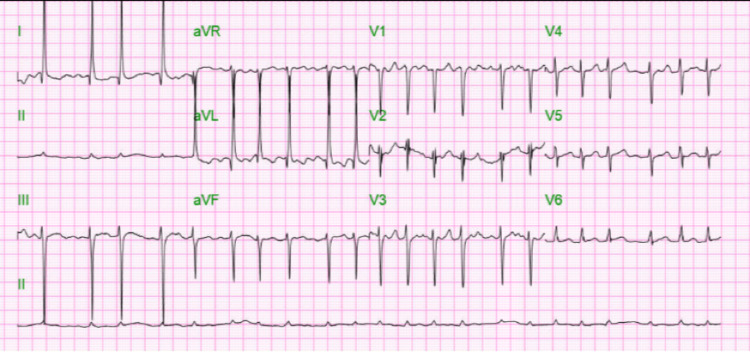
EKG on Admission EKG reveals atrial fibrillation with rapid ventricular response with a heart rate of 133 beats per minute.

Our patient is a 72-year-old female with a past medical history of diabetes mellitus and chronic kidney disease stage 3b, who was brought in by ambulance to the emergency department with altered mental status. According to the family, the patient was independent in activities of daily living prior to this admission. A physical exam on admission showed disorientation, dehydration, wheezing, and tachycardia. Remarkable labs on admission can be found in the table below (Table 1). The patient was found to be COVID-19 positive with bilateral pneumonia and ground glass opacities seen on chest X-ray (Figure 1) and chest CT (computerized tomography) scan (Figure 2). The patient was also in septic shock with tachycardia, tachypnea, leukocytosis, and elevated lactate. CT scan of the head was negative for any acute disease. Urine toxicology was also negative. Her electrocardiogram (EKG) on admission showed new-onset atrial fibrillation with a rapid ventricular response (Figure 3); she had no known previous history of atrial fibrillation. On admission, high-sensitivity troponins were elevated to greater than 20,000 nanograms per liter but no ST deviations or T-wave abnormalities suggesting acute ischemia were seen on EKG.

**Table 1:** Patient’s Laboratory Findings on Admission

Laboratory Test	Normal Range	Results
White Blood Cells	4,500 - 11,000 cells per microliter	13,500 cells per microliter
Hemoglobin	11.0 - 15.0 grams per deciliter	17.4 grams per deciliter
Hematocrit	35 - 46 percent	52.4 percent
Mean Corpuscular Volume	80 - 100 femtoliters	85.8 femtoliters
Platelets	130,000 - 400,000 platelets per microliter	111,000 platelets per microliter
Blood Urea Nitrogen	9.8 - 20.1 milligrams per deciliter	104.1 milligrams per deciliter
Creatinine	0.57 - 1.11 milligrams per deciliter	3.19 milligrams per deciliter
Estimated Glomerular Filtration Rate	>=90.0 millimeters per minute per 1.73 square meters	14.2 millimeters per minute per 1.73 square meters
Potassium	3.5 - 5.1 millimoles per liter	4.2 millimoles per liter
Phosphorus	2.3 - 4.7 milligrams per deciliter	4.0 milligrams per deciliter
Magnesium	1.6 - 2.6 milligrams per deciliter	3.3 milligrams per deciliter
D-dimer	<=500 nanograms per milliliter d-dimer units	> 7,650 nanograms per milliliter d-dimer units
Prothrombin time	9.8 - 13.4 seconds	15.5 seconds
International Normalized Ratio	0.85 - 1.15 ratio	1.27 ratio
Partial Thromboplastin Time	24.9 - 35.9 seconds	29.9 seconds
Thyroid Stimulating Hormone	0.465 - 4.680 micro-international units per milliliter	0.926 micro-international units per milliliter
Thyroxine	0.78 - 2.19 nanograms per deciliter	1.19 nanograms per deciliter
Brain Natriuretic Peptide	10.0 - 100.0 picograms per milliliter	365.2 picograms per milliliter

**Figure 4:**
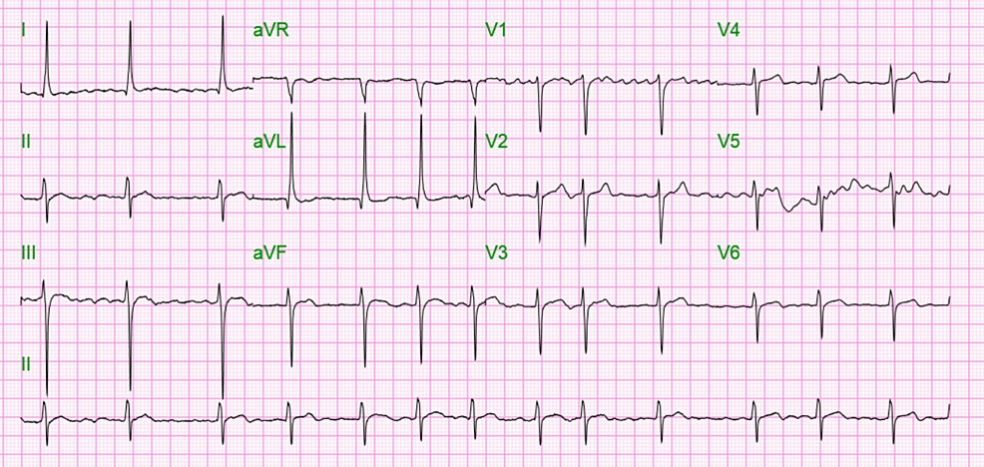
EKG Post-Cardizem Drip EKG shows atrial fibrillation with a controlled heart rate of 79 beats per minute.

The patient was treated with cardizem drip which was eventually switched to oral amiodarone and metoprolol; serial EKGs showed sustained atrial fibrillation with a controlled ventricular rate (Figure 4). The patient was medically managed for type 2 myocardial infarction. Cardiac catheterization and echocardiogram could not be done due to the patient’s unstable condition, but in view of the patient’s quick drop in troponin (Table 2) type 2 myocardial infarction is extremely likely. On day three, the patient began to desaturate to 80-85% and was using accessory muscles for respiration and so was intubated and placed on mechanical ventilation. The patient’s sputum culture also began growing Klebsiella pneumoniae extended-spectrum beta-lactamase which was treated with gentamicin. Dexamethasone was started for COVID-19 on admission. The patient’s neurological exam and brainstem reflexes were normal, and improvement was noted in the patient's respiratory parameters on a spontaneous breathing trial. A weaning trial off the ventilator was thus attempted but failed, and a tracheostomy was ultimately performed on day 21 of hospitalization. Percutaneous endoscopic gastrostomy feeding tube placement was planned, but the patient coded and died on day 23 of hospitalization.

**Table 2:** Troponin Trend

	On admission	Hour 8	Hour 16	Day 2	Day 7
High Sensitivity Troponin (Normal Range: 0.0 - 17.0 nanograms per liter)	20,334.6 nanograms per liter	21,049.0 nanograms per liter	21,250.0 nanograms per liter	2,817.3 nanograms per liter	799.1 nanograms per liter

## Discussion

Infection with the COVID-19 virus has been associated with adverse cardiovascular outcomes including arrhythmias, myocarditis, and myocardial infarction. It has been linked with the development of new-onset atrial fibrillation and with a poor prognosis [3]. These effects may be attributed to the expression of angiotensin-converting enzyme 2 (ACE-2) receptors on the myocardium [5]. Atrial fibrillation has been more prominently observed in patients with predisposing chronic cardiovascular diseases such as hypertension [2]. Other associated risk factors include diabetes, older age, heart failure, and renal impairment [2, 6]. 

There are several factors that have been presumed to play a role in the progression of new-onset atrial fibrillation. These factors include the involvement of ACE-2 receptors, inflammatory cytokine storm, electrolyte abnormalities, direct endothelial damage, and acid-base disorders [5, 7]. Additionally, a correlation has been observed between disease severity and the development of new-onset atrial fibrillation [3]. There is an increased incidence of AFIB in patients who experience renal deterioration, thrombotic complications, hypoxia, the need for critical care, and a longer length of hospital stay. The risk of new-onset AFIB has also been associated with an increased level of epicardial adipose tissue [8].

In this case, the patient developed acute myocardial infarction with new-onset atrial fibrillation during acute COVID-19 infection. Our patient also had sepsis which could have triggered or contributed to the development of atrial fibrillation. In a study based on the American Heart Association’s COVID-19 cardiovascular registry, the incidence of new-onset AFIB was 5.4% [3]. Among those with new-onset AFIB, the incidence of myocardial infarction was 9.8% while the incidence of myocardial infarction was 2.7% in COVID-19 patients without AFIB. An association was found between new-onset atrial fibrillation and major adverse cardiovascular events such as myocardial infarction. 

New-onset atrial fibrillation has been associated with worsening clinical outcomes in COVID-19 patients [9]. It is a life-threatening complication that is associated with a two-fold increase in mortality and a three-fold increase in secondary outcomes. Secondary outcomes due to new-onset AFIB include major adverse cardiovascular events such as cardiovascular death, myocardial infarction, new-onset heart failure, stroke, and cardiogenic shock. Other adverse outcomes include the need for critical care, mechanical ventilation, vasopressors, renal replacement therapy, and thromboembolic complications. New-onset AFIB has been associated with a higher incidence of embolic events [10]. 

Management of new-onset atrial fibrillation in COVID-19 patients is focused on rate control [11]. The use of beta-blockers and non-dihydropyridine calcium channel blockers is the mainstay for long-term management after stabilization. In patients with acute decompensation, intravenous digoxin or amiodarone is used for rate control and stabilization [12]. The use of anticoagulation with warfarin, novel oral anticoagulants, or low molecular weight heparin based on risk factors is indicated to reduce thromboembolic risk [13].

## Conclusions

Here we present a case of new-onset atrial fibrillation and type 2 myocardial infarction in a patient that presented with COVID-19 pneumonia. To our knowledge, this is the first case that documented both new-onset atrial fibrillation and acute myocardial infarction in a single patient with COVID-19 pneumonia. It is important to be on the lookout for cardiovascular complications, like atrial fibrillation and myocardial infarction in patients presenting with COVID-19.
